# The Selective Separation of Carnosic Acid and Rosmarinic Acid by Solid-Phase Extraction and Liquid–Liquid Extraction: A Comparative Study

**DOI:** 10.3390/molecules28145493

**Published:** 2023-07-18

**Authors:** Chunyan Zhu, Yunchang Fan, Hongwei Wu

**Affiliations:** 1 College of Chemistry and Chemical Engineering, Henan Polytechnic University, Jiaozuo 454003, China; 212012020034@home.hpu.edu.cn; 2 Department of Chemistry, Xinxiang Medical University, Xinxiang 453003, China

**Keywords:** *Rosmarinus officinalis* leaves (ROLs), carnosic acid (CA), rosmarinic acid (RA), macroporous resin, solid-phase extraction (SPE), liquid–liquid extraction (LLE)

## Abstract

*Rosmarinus officinalis* leaves (ROLs) are widely used in the food and cosmetics industries due to their high antioxidant activity and fascinating flavor properties. Carnosic acid (CA) and rosmarinic acid (RA) are regarded as the characteristic antioxidant components of ROLs, and the selective separation of CA and RA remains a significant challenge. In this work, the feasibility of achieving the selective separation of CA and RA from ROLs by solid-phase extraction (SPE) and liquid–liquid extraction (LLE) was studied and compared. The experiments suggested that SPE with CAD-40 macroporous resin as the adsorbent was a good choice for selectively isolating CA from the extracts of ROLs and could produce raw CA with purity levels as high as 76.5%. The LLE with ethyl acetate (EA) as the extraction solvent was more suitable for extracting RA from the diluted extracts of ROLs and could produce raw RA with a purity level of 56.3%. Compared with the reported column chromatography and LLE techniques, the developed SPE–LLE method not only exhibited higher extraction efficiency for CA and RA, but can also produce CA and RA with higher purity.

## 1. Introduction

*Rosmarinus officinalis* leaves (ROLs) are highly commercialized plants and are known for their compounds that are rich in antioxidants, such as CA and RA [1]. The excellent biological activities of ROLs can be attributed to the anti-inflammatory, antibacterial, anticancer, and antiviral properties of these two compounds, making ROLs suitable to be widely used as food antioxidants, anti-aging cosmetics, natural food additives, health products, and medicines [2,3]. Therefore, the isolation of CA and RA from ROLs has become a hot research topic and a variety of techniques, including supercritical fluid extraction (SFE) [4], column chromatographic extraction (CCE) [5], LLE [6,7], and column chromatography (CC) [8,9], have been developed for this purpose. For example, Lefebvre et al. developed an online SFE-supercritical fluid chromatography (SFC) system using a mixture of carbon dioxide, ethanol, and water as the solvents to isolate CA and RA from rosemary; high-purity CA (49%) and RA (78%) could be obtained by adjusting the polarity of the solvents [4]. Zhu et al. successfully obtained an RA extract with strong biological activity from *Perilla* leaves by using the supramolecular formation technique coupled with LLE (supramolecular formation-LLE) using EA as the extraction solvent; a supernatant containing RA was isolated from the crude extract of the *Perilla* leaf through supramolecular formation and was then evaporated to dryness. The resultant residue was dissolved in a 1% trifluoroacetic acid aqueous solution, followed by the addition of EA for the extraction of the RA. After evaporating the EA under vacuum conditions, an RA extract with levels of purity in the range of 58.4% to 67.4% was obtained [6]. Wang et al. used LLE composed of choline chloride (ChCl)-levulinic acid (LA)/1-butyl-3-methylimidazolium hexafluorophosphate ([C_4_mim]PF_6_)/H_2_O to extract CA and RA from ROLs. The suggested LLE exhibited high extraction efficiency for CA (97.46%) and RA (88.97%) [7].

Kim et al. suggested a CC method using the epichlorohydrin-crosslinked dextran gel, Sephadex LH-20, as the stationary phase and 100% methanol as the eluent to purify RA from the extract (10% aqueous methanol) of lemon balm leaves; RA with a purity of 38.8% could be produced [8]. Zhang and coworkers also adopted the CC method with NK-109 macroporous resin as the stationary phase to purify RA from the extract of *Perilla* seeds;they found that the adsorption of RA on the NK-109 macroporous resin was in good agreement with the pseudo-second-order kinetic and Langmuir adsorption models. After conducting desorption using methanol as eluent, a crude extract containing 42.1% of RA could be obtained [9].

Based on the above discussion, it should be noted that, although SFE-SFC is an effective technique for producing high-purity CA and RA, it is an expensive method [10]. The supramolecular formation-LLE can produce high-purity RA (58.4% to 67.4%), but the feasibility of this method for the purification of CA is not mentioned [6]; ChCl-LA/[C_4_mim]PF_6_/H_2_O based LLE exhibits high extraction efficiency for CA and RA, but the further purification of CA and RA is not discussed in their work [7].Furthermore, CC methods with Sephadex LH-20 gel and NK-109 macroporous resin as the stationary phases can produce only low-purity RA (38.8% to 42.1%) [8,9]. Therefore, the development of a cost-effective method for the selective extraction and purification of RA and CA is of great importance. Recently, LLE with green solvents, ionic liquids (ILs), and biodegradable natural deep eutectic solvents (BNDESs) as extraction solvents has been used for the extraction of bioactive compounds from natural sources [7]. Meanwhile, SPE is also widely used to extract and purify bioactive compounds from natural sources due to its advantages of low costs, simple operations, and high efficiency [11,12].

Therefore, the aims of this work are to investigate the feasibility of the coupling of SPE and LLE for the selective extraction and purification of RA and CA from the extracts of ROLs (45% aqueous polyethylene glycol 400 (PEG-400)) [13] and to compare the advantages and disadvantages of SPE with macroporous resin as the adsorbent and LLE with ILs, BNDESs, and the conventional solvent EA as the extraction solvents. Finally, the BNDESs composed of *L*-menthyl lactate (ML) and butyl lactate (BL) (referred to as ML-BL) are chosen as the extraction solvents due to their biodegradable and low-toxic nature [14,15].

## 2. Results and Discussion

### 2.1. Identification of Hydrogen Bond Donors (HBDs) and Hydrogen Bond Acceptors (HBAs) in BNDESs

**Figure 1:**
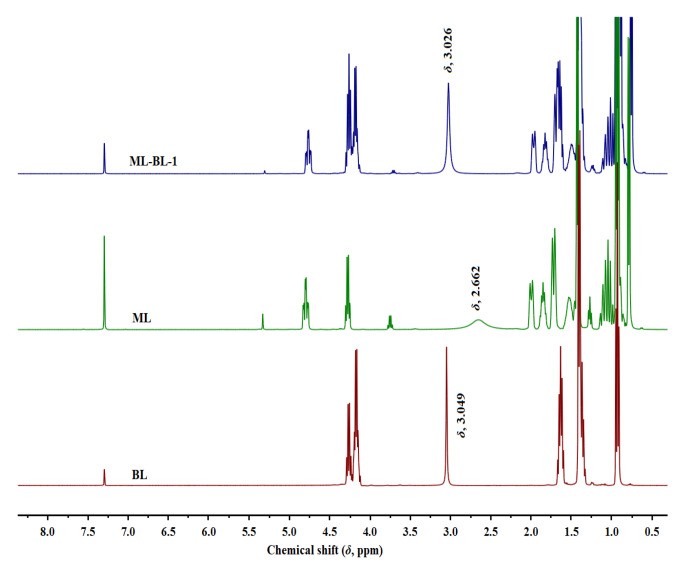
The ^1^H NMR spectra of BL, ML, and ML-BL-1 (400 MHz, CDCl_3_). The chemical shifts marked in the figure are the hydroxyl proton signals of the corresponding compounds.

In this work, four ML-BL-based BNDESs were prepared via the hydrogen bond between ML and BL; their proton nuclear magnetic resonance (^1^H NMR) and Fourier transform infrared (FT-IR) spectra are shown in Figures S1–S8 in the Supplementary Materials. To identify HBDs and HBAs in BNDESs, the ^1^H NMR of BNDESs and the corresponding raw materials were measured and the results are illustrated in Figure 1 and Figures S9–S11. The results indicate that, after mixing with each other, the chemical shifts of the hydroxyl protons of BL and ML overlap; the chemical shifts of the hydroxyl protons of BL in the ML-BL-based BNDESs shift to lower values compared with the pure BL, and the opposite phenomenon can be observed for ML, suggesting that BL and ML act as HBAs and HBDs, respectively [16,17].

### 2.2. Separation and Purification of CA and RA from the PEG-400 Extract

**Figure 2:**
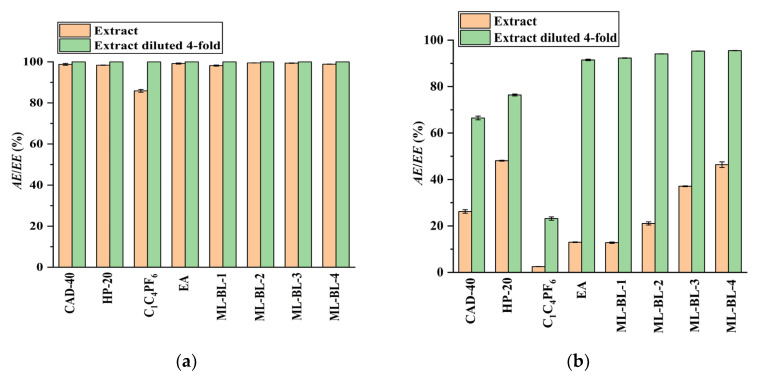
Comparison of the extraction/adsorption of CA (**a**) and RA (**b**) from the PEG-400 extract and the PEG-400 extract diluted four-fold; *AE*: adsorption efficiency; *EE*: extraction efficiency. Experimental conditions: solid–liquid ratio for CAD-40 and HP-20, 0.2 g mL^−1^; the phase volume ratio for the extraction of CA and RA by EA, [C_4_mim]PF_6_, ML-BL-1, ML-BL-2, ML-BL-3, and ML-BL-4 was 1:1.

**Figure 3:**
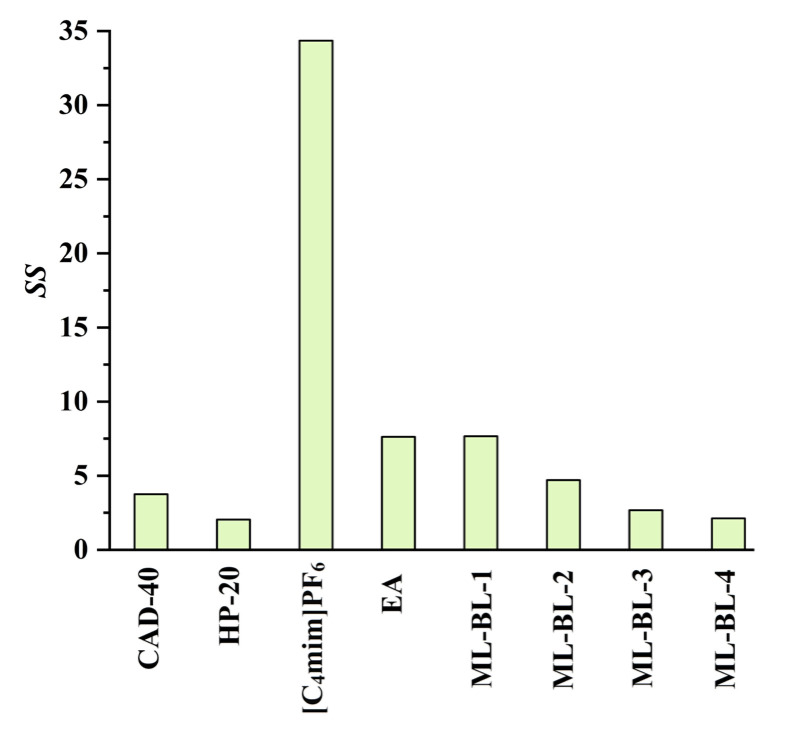
Separation selectivity (*SS* values) of CAD-40, HP-20, [C_4_mim]PF_6_, EA, ML-BL-1, ML-BL-2, ML-BL-3, and ML-BL-4; *SS*: the ratio of the *AE* value of CA to that of RA or the ratio of the *EE* value of CA to that of RA.

In the present work, the feasibility of using the SPE and LLE methods to recover CA and RA from the PEG-400 extract was studied (the optimization of the SPE and LLE conditions is demonstrated in Figures S12–S17 in Section S3 in the Supplementary Materials). Macroporous resins including CAD-40 (a polar resin) and HP-20 (a non-polar resin) were selected as adsorbents due to their strong adsorption ability for organic acids [18,19]. As a comparison to SPE, the extraction ability of LLE with the IL [C_4_mim]PF_6_ [7], ML-BL-based BNDESs (their physicochemical properties are listed in Table 1; the molar ratios of ML to BL for ML-BL-1 to ML-BL-4 are 1:1, 1:2, 1:5, and 1:10, respectively) and EA [20,21] as extraction solvents is also investigated. The experimental results illustrated in Figure 2 show that both the SPE and LLE methods exhibit high extraction/adsorption abilities for CA but poor extraction/adsorption abilities for RA, referring to the feasibility of the selective recovery of CA from the extract. As shown in Figure 3, [C_4_mim]PF_6_, EA, ML-BL-1, and CAD-40 possess high separation selectivity (*SS*) for CA. However, [C_4_mim]PF_6_ and ML-BL-1 are high-boiling-point solvents; as such, it is difficult to remove them via distillation after extraction. To recover CA from [C_4_mim]PF_6_ and ML-BL-1, back-extraction using a phosphate buffer (pH 7, 0.1 mol L^−1^) or an ammonia buffer (pH 9, 0.1 mol L^−1^) as solvents was conducted; the experimental results suggest that no CA is found in the two back-extraction solvents, indicating that [C_4_mim]PF_6_ and ML-BL-1 are not suitable for the recovery of CA from the PEG-400 extract.

Based on the above observations, it seems that EA and CAD-40 are more suitable for recovering CA from the PEG-400 extract. When using CAD-40 to recover CA from the PEG-400 extract, a solid–liquid ratio of 0.1 g mL^–1^ is used (Figures S13b and S14b in the Supplementary Materials).

### 2.3. Adsorption Isotherms, Kinetics, and Thermodynamics of CA and RA on CAD-40

The adsorption behaviors of CA and RA on CAD-40 were first studied using the Langmuir and Freundlich models, respectively [22,23]:

(1) $$\mathrm{Langmuir}\;\mathrm{equation}:\;\frac{1}{q_{e}}=\frac{1}{q_{m}K_{L}}\frac{1}{C_{e}}+\frac{1}{q_{m}}$$

(2) $$\mathrm{Freundlich}\;\mathrm{equation}:\;\ln q_{e}=\ln K_{F}+\frac{1}{n}\ln C_{e}$$

where *q*_e_, *q*_m_, *K*_L_, *C*_e_, *K*_F_, and 1/*n* refer to the equilibrium adsorption amount (mg g^−1^), the maximum adsorption amount (mg g^−1^), the Langmuir constant (L mg^−1^), the solute equilibrium concentration (mg L^−1^), the Freundlich constant (mg g^–1^ (L mg^−1^)^1/*n*^), and an empirical constant related to the adsorption capacity, respectively.

**Figure 4:**
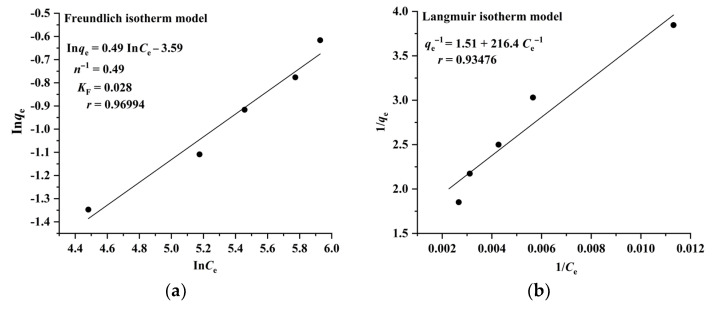
Fitting results of the Freundlich (**a**) and Langmuir isotherm (**b**) models for the adsorption of RA on CAD-40 macroporous resin from the PEG-400 extract; *r*: correlation coefficient.

**Figure 5:**
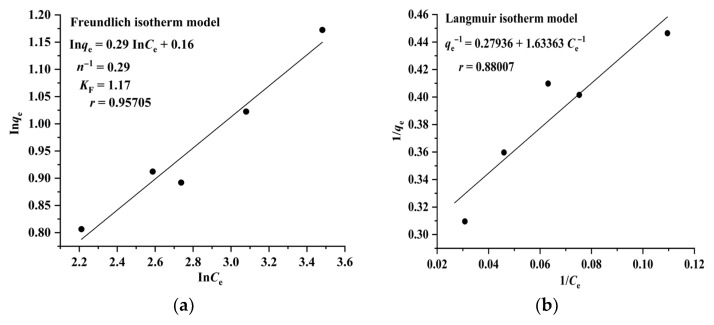
Fitting results of the Freundlich (**a**) and Langmuir (**b**) isotherm models for the adsorption of CA on CAD-40 macroporous resin from the PEG-400 extract.

The Langmuir model assumes that adsorption takes place on a homogeneous surface and is a monolayer molecular adsorption process, whereas the Freundlich model suggests that adsorption occurs on a heterogeneous surface and is a multilayer adsorption process. The results illustrated in Figure 4 and Figure 5 indicate that the adsorption of RA and CA on CAD-40 correlates well with the Freundlich model (*r* = 0.96994 for RA (Figure 4a); *r*= 0.95705 for CA (Figure 5a)) compared with the Langmuir equation (*r*= 0.93476 for RA (Figure 4b); *r* = 0.88007 for CA (Figure 5b)).

Additionally, the adsorption kinetics of CA and RA on CAD-40 were also investigated using the pseudo-first-order and pseudo-second-order equations [24,25]:

(3) $$\mathrm{pseudo-first-order}\;\mathrm{equation}:\;\ln\;(q_{e}-q_{t})=\ln q_{e}-k_{1}t$$

(4) $$\mathrm{pseudo-second-order}\;\mathrm{equation}:\;\frac{t}{q_{t}}=\frac{1}{q_{e}^{2}k_{2}}+\frac{t}{q_{e}}$$

where *k*_1_ and *k*_2_ refer to the adsorption rate constants of the pseudo-first-order and pseudo-second-order, respectively.

**Figure 6:**
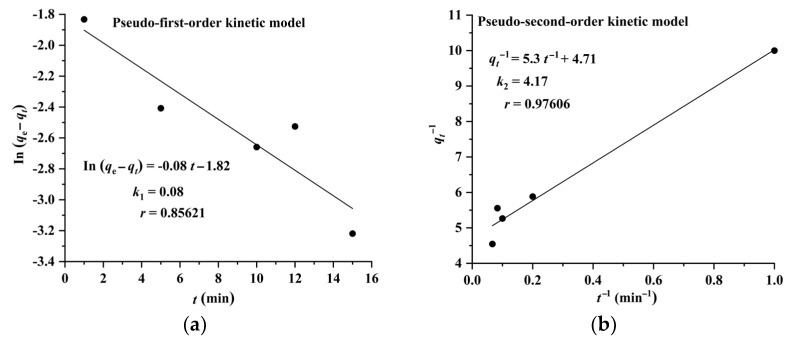
Plots of pseudo-first-order (**a**) and pseudo-second-order (**b**) kinetic models for the adsorption of RA on CAD-40 macroporous resin from the PEG-400 extract.

**Figure 7:**
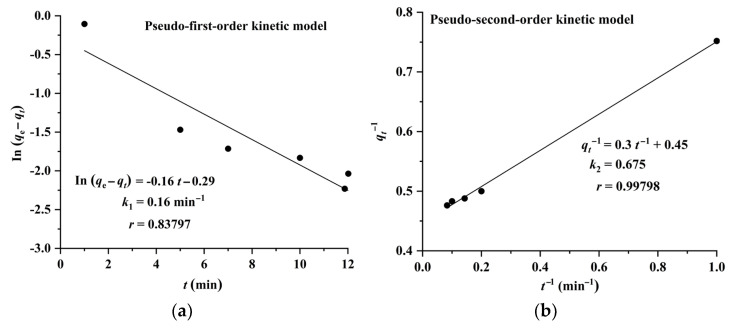
Plots of pseudo-first-order (**a**) and pseudo-second-order (**b**) kinetic models for the adsorption of CA on CAD-40 macroporous resin from the PEG-400 extract.

The results shown in Figure 6 and Figure 7 indicate that the adsorption of CA and RA on CAD-40 fits well with the pseudo-second-order model, compared with the pseudo-first-order equation.

In order to further investigate the adsorption mechanism, the thermodynamic parameters of the adsorption process, such as the Gibbs free energy change (Δ*G*), enthalpy change (Δ*H*), and entropy change (Δ*S*), are calculated using the following equations [26,27]:

(5) $$\ln\frac{q_{e}}{C_{e}}=\frac{\Delta S}{R}-\frac{\Delta H}{RT}$$

**Figure 8:**
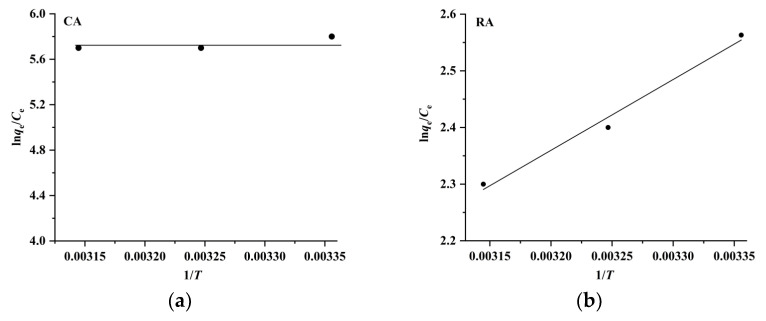
Plots of ln (*q*_e_/*C*_e_) versus 1/*T* for the adsorption of CA (**a**) and RA (**b**) on CAD-40 macroporous resin from the PEG-400 extract.

(6) ΔG=ΔH−TΔS

where R and *T* are the gas constant (8.314 J mol^−1^ K^−1^) and absolute temperature (K), respectively. The Δ*H* and Δ*S* values are obtained by plotting ln (*q*_e_/*C*_e_) versus 1/*T* (Figure 8), and the results listed in Table 2 indicate that the Δ*G* values are negative and in the range of –6.1 kJ mol^–1^ to –15.1 kJ mol^−1^, meaning that the adsorption of CA and RA on CAD-40 is spontaneous and dominated by physical adsorption (generally, the Δ*G* values for chemical adsorption are in the range of −80 kJ mol^−1^ to −400 kJ mol^−1^, while the Δ*G* values for physical adsorption range from −20 kJ mol^−1^ to 0 kJ mol^−1^ [27]). Meanwhile, the Δ*H* values for the adsorption of CA and RA are in the range of 0 kJ mol^−^^1^ to −10.4 kJ mol^−^^1^, indicating once again that the adsorption of CA and RA on CAD-40 may be a physical adsorption process (the Δ*H* value for chemical adsorption is in the range of 84 kJ mol^−^^1^ to 420 kJ mol^−^^1^ and that for physical adsorption is below 84 kJ mol^−^^1^ [28]).

**Figure 9:**
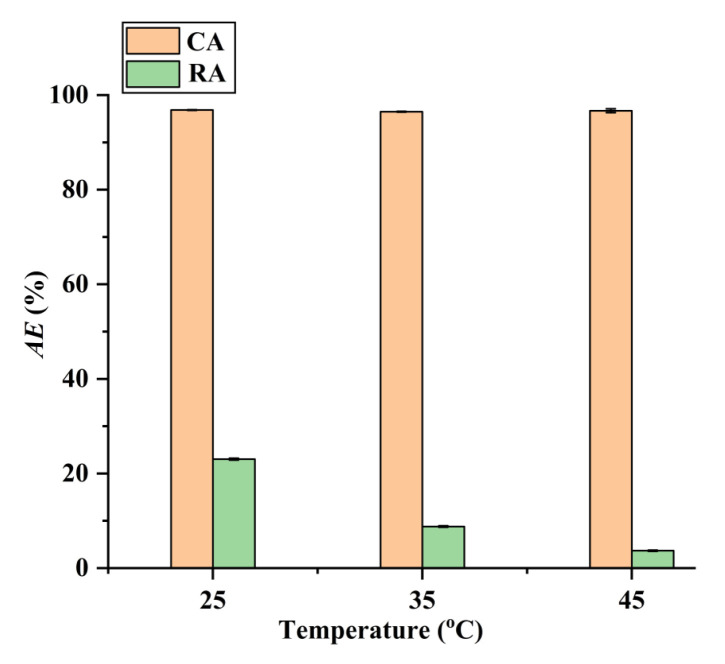
Effect of temperature on the adsorption of RA and CA on CAD-40 macroporous resin from the PEG-400 extract. Solid–liquid ratio, 0.1 g mL^−1^.

Additionally, as shown in Figure 9, the adsorption operating at higher temperatures can improve the adsorption selectivity for CA. Therefore, the selective separation of CA by the CAD-40 macroporous resin was conducted at 45 °C. After adsorption, the CAD-40 macroporous resin was washed with 95% (*v*/*v*) ethanol to recover CA with a desorption efficiency (*DE*) of 98.5%. After removing the ethanol via distillation under vacuum conditions, brown oil was obtained and 85% H_3_PO_4_ was added to the resultant oil to adjust the pH value to about 2, leading to the generation of a brown precipitate. This precipitate was washed with water and subjected to freeze-drying to obtain raw CA (purity: 76.8%, determined by high-performance liquid chromatography (HPLC) as described in Section 3.5).

When using EA as the extraction solvent, the isolation of CA from the PEG-400 extract was conducted at a phase volume ratio of 1:1 (*V*_EA_:*V*_PEG-400 extract_); after extraction, the EA was removed via vacuum distillation to obtain brown oil, and the resultant oil was acidified to a pH of about 2 using 85% H_3_PO_4_ to generate a brown precipitate. This precipitate was washed with water and vacuum freeze-dried to obtain raw CA (purity: 64.2%).

The above results suggest that the SPE method with CAD-40 macroporous resin as the adsorbent may be a better choice for the recovery of CA from the PEG-400 extract because it can provide higher-purity CA compared with LLE when using EA as extraction solvent.

As illustrated in Figure 2, both the CAD-40 and HP-20 macroporous resins exhibit low extraction efficiency for RA, even after diluting the PEG-400 extract four times with water to diminish the effect of PEG-400 (*AE*_RA_ < 80%); meanwhile, EA and the BNDES ML-BL-1 possess higher extraction efficiency for RA. When ML-BL-1 is used to recover RA from the PEG-400 extract with four-fold dilution, it is not feasible to separate the RA from the ML-BL-1 phase via distillation because ML-BL-1 has a high boiling point. Therefore, after extraction, the ML-BL-1 phase is washed using an ammonia buffer (pH 10, 0.1 mol L^−1^) to recover the RA (back-extraction efficiency: 94.6%); the resultant back-extraction solution is acidified to a pH of about 2 by 85% H_3_PO_4_ and then extracted by an equal volume of EA. After the removal of the EA via distillation, a light yellow liquid containing large amounts of ML-BL-1 and trace amounts of RA is obtained because ML-BL-based BNDESs are partially soluble in water (908.3 mg (100 mL)^−1^–1674.1 mg (100 mL)^−1^, Table 1), meaning that it is difficult to separate the RA from ML-BL-based BNDESs when using ML-BL-based BNDESs as extraction solvents. In contrast, EA may be a better choice because it possesses high extraction efficiency for RA (Figure 2) and has a low boiling point. When EA is used as extraction solvent, the extraction of RA from the PEG-400 extract with four-fold dilution is conducted at a phase volume ratio of 1:1; the EA is removed via vacuum distillation and a brown oil is generated. This brown oil is washed with an ammonia buffer (pH 10, 0.1 mol L^−1^), the resultant solution is acidified to a pH of about 2 by 85% H_3_PO_4_ and it is then extracted by EA at a phase volume ratio of 1:1. After the removal of EA via vacuum distillation, raw RA is obtained as a brown powder with a purity of 56.3%.

### 2.4. Comparison with the Reported Methods

Recently, various methods, such as CCE [5], supramolecular formation-LLE using EA as the extraction solvent [6], LLE composed of ChCl-LA/[C_4_mim]PF_6_/H_2_O [7], CC with Sephadex LH-20 gel [8], and NK-109 macroporous resin [9] as the stationary phases, have been used for the purification of RA and CA. A comparison between the SPE-LLE method suggested by this work and the reported techniques for the extraction/adsorption and purification of RA and CA was thus conducted, and the results listed in Table 3 indicate that, compared with the reported methods, the suggested SPE-LLE technique exhibits higher extraction/adsorption abilities for RA and CA. Meanwhile, the CA obtained by the suggested SPE-LLE method has the highest purity. The purity of the RA obtained by the suggested SPE-LLE method is close to that produced by the supramolecular formation-LLE technique and higher than that obtained by the remaining reported methods.

## 3. Materials and Methods

### 3.1. Reagents and Materials

Chemicals including CA (≥97%), RA (≥97%), BL (98%), and ML (97%) were purchased from Aladdin Biochemical Technology Co., Ltd. (Shanghai, China). Polyethylene glycol (number average molecular weight, 400) (PEG-400) was supplied by Yantian Biotechnology Co., Ltd. (Shanghai, China). The IL, [C_4_mim]PF_6_ (99%) was obtained from the Lanzhou Institute of Chemical Physics of the Chinese Academy of Sciences (Lanzhou, China). Reichardt’s dye (RD, 90%) and 4-nitroaniline (NA, ≥99%) were supplied by Sigma-Aldrich Co., (St. Louis, MO, USA); *N*,*N*-diethyl-4-nitroaniline (DENA, 97%) was obtained from Fluorochem Ltd., (Hadfield, UK). The macroporous resins CAD-40 (polar resin, specific surface area: 450–500 m^2^ g^−1^, particle size: 60–16 meshes) and HP-20 (non-polar resin, specific surface area: 550–600 m^2^ g^−1^, particle size: 60–16 meshes) were supplied by Xinhu Laboratory Equipment Co., Ltd. (Shanghai, China). All the other reagents are of analytical grade and were used as received. Dried ROLs were supplied by Haoruijia Biotechnology Co., Ltd. (Chengdu, China) and crushed into fine powers (100 meshes) before use. The extract of the ROLs was prepared using 45% PEG-400 as the extraction solvent containing 4.3% H_3_PO_4_ [13].

### 3.2. Synthesis of BNDESs

Four BNDESs composed of ML and BL were synthesized by mixing ML and BL together at different mole ratios at 60 °C until homogeneous and colorless liquids were formed. The four BNDESs are denoted as ML-BL-1 (ML:BL = 1:1), ML-BL-2 (ML:BL = 1:2), ML-BL-3 (ML:BL = 1:5), and ML-BL-4 (ML:BL = 1:10). Their chemical structures were characterized by ^1^H NMR and FT-IR spectroscopy (Figures S1–S8 in the Supplementary Materials).

### 3.3. Measurements of the Viscosities and Solubilities of the Synthesized BNDESs

The viscosities of the synthesized BNDESs were measured using a rotary viscometer (model NDJ-1F, Changji Geological Instrument Co., Ltd., Shanghai, China).

The solubilities of the BNDESs were determined by mixing equal volumes of a given BNDES and water by stirring for 30 min at 25 °C; then, the resultant mixture was left for 24 h to achieve phase equilibrium. The concentrations of the BNDESs in water were analyzed using an Agilent 1200 HPLC (Agilent Technologies, Santa Clara, CA, USA) and the chromatographic separation was conducted on an Amethyst C18-H column (4.6 mm × 150 mm, 5 μm, Sepax Technologies Inc., Newark, DE, USA); the column temperature was 30 °C. The mobile phase constituted a mixture of 60% (*v*/*v*) acetonitrile and 40% water containing 0.1% acetic acid with a flow rate of 1.0 mL min^−1^. The detection wavelength was 220 nm and the injection volume was 5.0 μL.

### 3.4. Determination of the Kamlet–Taft Polarity Parameters of the Synthesized BNDESs

The hydrogen bonding parameters of the prepared BNDESs, including *α* and *β*, were measured using RD, NA, and DENA as probes according to the reported methods [29,30]. The concentrations of RD, NA, and DENA in the BNDESs were 8.0 × 10^−4^ mol L^−1^, 3.3 × 10^−5^ mol L^−1^, and 8.3 × 10^−5^ mol L^−1^, respectively. The maximum absorption wavelengths (λ_max_) of the three dyes in the BNDESs were determined using a spectrophotometer (TU-1810, Persee Co., Beijing, China). The hydrogen bonding parameters, *α* and *β*, were calculated using the following equations [29,30]:

(7) $$\nu_{\max}({\mathrm{cm}}^{-1})=\frac{10^{4}}{\lambda_{\max}\;(\mathrm{nm})}$$

(8) $$E_{T}(30)=28592/\lambda \max(\mathrm{RD})$$

(9) $$\pi^{*}=0.314(27.52-\nu_{\max}(\mathrm{DENA}))$$

(10) $$\alpha =0.0649E_{T}(30)-2.03-(0.72\pi^{*})$$

(11) $$\beta =(1.035\nu_{\max}(\mathrm{DENA})+2.64-\nu_{\max}(\mathrm{NA}))/2.8$$

where *ν*, *E*_T_(30), and π* are the wavenumbers corresponding to the maximum absorption wavelength, solvent polarity, and solvent dipolarity/polarisability, respectively.

### 3.5. Determination of CA and RA

The determination of CA and RA was performed by HPLC under the following conditions: separation column, Amethyst C18-H column; mobile phase, the mixture of acetonitrile and 0.1% (*v*/*v*) acetic acid aqueous solution, 65% (*v*/*v*) of acetonitrile with a flow rate of 1.0 mL min^−1^ was used for the determination of CA and 20% acetonitrile with a flow rate of 0.85 mL min^−1^ was adopted for the analysis of RA; a detection wavelength of 330 nm was set for the analysis of RA and 250 nm was used for the determination of CA; column temperature, 30 °C; injection volume, 5.0 μL.

### 3.6. Adsorption of CA and RA on Macroporous Resins

Generally, 1.0 g of macroporous resin and 10 mL of the PEG-400 extract were mixed by stirring at a given temperature and the concentrations of CA and RA in the adsorption process at different time intervals were measured using the HPLC methods. The adsorption efficiency (*AE*), adsorption amount (*q*), and desorption efficiency (*DE*) were calculated using the following equations:

(12) $$AE\%=\frac{C_{0}-C_{t}}{C_{0}}\times 100$$

(13) $$q_{e,t}=\frac{V(C_{0}-C_{e,t})}{m}$$

(14) $$DE\%=\frac{C_{d}V_{d}}{V(C_{0}-C_{e})}\times 100$$

where *C*_0_, *C_e_*_,_ and *C_t_* refer to the initial solute (CA or RA) concentration (mg L^−1^), the solute concentration after achieving adsorption equilibrium, and that at a given time (*t*, min) respectively; *q*_e,*t*_ (mg g^−1^) is the adsorption amount of CA or RA at equilibrium (*q*_e_) or at a given time (*q_t_*), *V* refers to the volume of the PEG-400 extract (L), *m* is the mass of the macroporous resin (g), *C*_d_ refers to the solute concentration in the desorption solution, and *V*_d_ is the volume of the desorption solution (L).

The separation selectivity (*SS*) of SPE and LLE for CA and RA was defined as the ratio of the *AE* (or *EE*) value of CA to that of RA:

(15) $$\mathrm{SS}=\frac{{\mathrm{AE}}_{\mathrm{CA}}(\mathrm{or}{\mathrm{EE}}_{\mathrm{CA}})}{{\mathrm{AE}}_{\mathrm{RA}}(\mathrm{or}{\mathrm{EE}}_{\mathrm{RA}})}$$

Raw CA and RA were dried in an LGJ-10 vacuum freeze-dryer (Beijing Songyuan Huaxing Technology development Co., Ltd., Beijing, China). All the experiments were conducted in duplicate and the data are expressed as means ± standard deviations (SDs).

## 4. Conclusions

In this work, the advantages and disadvantages of using LLE and SPE for the extraction/adsorption of CA and RA from PEG-400 extracts were systematically investigated. The experimental results indicated that, although the LLE with ML-BL-based BNDESs as extraction solvents possessed higher extraction efficiencies for CA (>90%), it was difficult to separate CA from the ML-BL phase via back-extraction and distillation, and the use of the IL [C_4_mim]PF_6_ as the extraction solvent has the same problem. The SPE with the CAD-40 macroporous resin as the adsorbent could selectively isolate CA from the extract of ROLs due to its strong adsorption ability for CA and poor adsorption ability for RA. The adsorption of CA and RA on the CAD-40 macroporous resin followed the Freundlich and pseudo-second-order models and was a physical adsorption process. The LLE with EA as the extraction solvent was a better choice for recovering RA from the diluted extracts of ROLs.

## Figures and Tables

**Table 1:** The viscosities, solubilities, hydrogen-bond-donating ability (*α*), hydrogen-bond-accepting ability (*β*), and *αβ* values of the synthesized ML-BL-based BNDESs (mean ± SD, *n* = 2).

BNDES	Viscosity (mPa⋅s)	Solubility in Water (mg (100 mL)^−1^)	*α*	*β*	*αβ*
ML-BL-1	23.4 ± 0.21	908.3 ± 1.3	0.69 ± 0.011	0.64 ± 0.0086	0.44 ± 0.013
ML-BL-2	12.4 ± 0.071	1142.7 ± 0.26	0.72 ± 0.0039	0.64 ± 0.00	0.46 ± 0.0025
ML-BL-3	6.2 ± 0.014	1603.9 ± 0.064	0.74 ± 0.010	0.64 ± 0.017	0.47 ± 0.019
ML-BL-4	5.0 ± 0.0071	1674.1 ± 0.064	0.74 ± 0.0052	0.65 ± 0.0086	0.48 ± 0.0097

**Table 2:** Thermodynamic parameters for the adsorption of CA and RA on CAD-40 macroporous resin.

Target Compound	*T* (K)	∆*G* (kJ mol^–1^)	∆*H* (kJ mol^–1^)	∆*S* (J mol^–1^ K^–1^)
CA	288	−14.1	0	47.4
	298	−14.6		
	308	−15.1		
RA	288	−6.3	−10.4	−13.6
	298	−6.2		
	308	−6.1		

**Table 3:** Comparison of different methods for the extraction/adsorption of RA and CA.

Separation Method	Extraction/Adsorption Efficiency	Purity	Reference
CCE	96% for CA and 94% for RA	6.84% for RA; 31.18% for CA	[5]
Supramolecular formation-LLE	– ^a^	58.4–67.4% for RA	[6]
LLE composed of ChCl-LA/[C_4_mim]PF_6_/H_2_O	97.46% for CA; 88.97% for RA	–	[7]
CC with Sephadex LH-20 as the stationary phase	–	38.8% for RA	[8]
CC with NK-109 macroporous resin as the stationary phase	68.3% for RA	42.1% for RA	[9]
SPE with CAD-40 macroporous resin as the adsorbent coupled with LLE, using EA as the extraction solvent	98.8% for CA; 91.5% for RA	76.8% for CA; 56.3% for RA	This work

^a^ Not mentioned.

## Data Availability

Not applicable.

## Supplementary Materials

The following supporting information can be downloaded at: https://www.mdpi.com/article/10.3390/molecules28145493/s1, Figures S1–S8: The ^1^H NMR and FT-IR spectra of the prepared BNDESs; Figures S9–S11: ^1^H NMR spectra of BL, ML, ML-BL-2, ML-BL-3 and ML-BL-4; Figures S12–S17: Optimization of the SPE and LLE conditions.

## Sample Availability

Samples of the extract of ROLs, PEG-400 extract, raw CA and RA are available from the authors.
