# Supplementary material for: The Selective Separation of Carnosic Acid and Rosmarinic Acid by Solid-Phase Extraction and Liquid–Liquid Extraction: A Comparative Study
Source: Molecules. 2023 Jul 18;28(14):5493. doi: 10.3390/molecules28145493 (PMC10386460; doi:10.3390/molecules28145493)
Supplement: Supplementary file 1 [file molecules-28-05493-s001.zip › molecules-2470205-supplementary.pdf]

The Selective Separation of Carnosic Acid and Rosmarinic Acid by Solid-Phase Extraction and Liquid–Liquid Extraction: A Comparative Study

Chunyan Zhu[1], Yunchang Fan[1,]* and Hongwei Wu[2,]*

[1]College of Chemistry and Chemical Engineering, Henan Polytechnic University, Jiaozuo 454003, China; 212012020034@home.hpu.edu.cn

[2]Department of Chemistry, Xinxiang Medical University, Xinxiang 453003 China

*Correspondence: fanyunchang@hpu.edu.cn (Y.F.); 011026@xxmu.edu.cn (H. W.)

Section S1. The ^{1}H NMR and FT-IR Spectra of the Prepared BNDESs

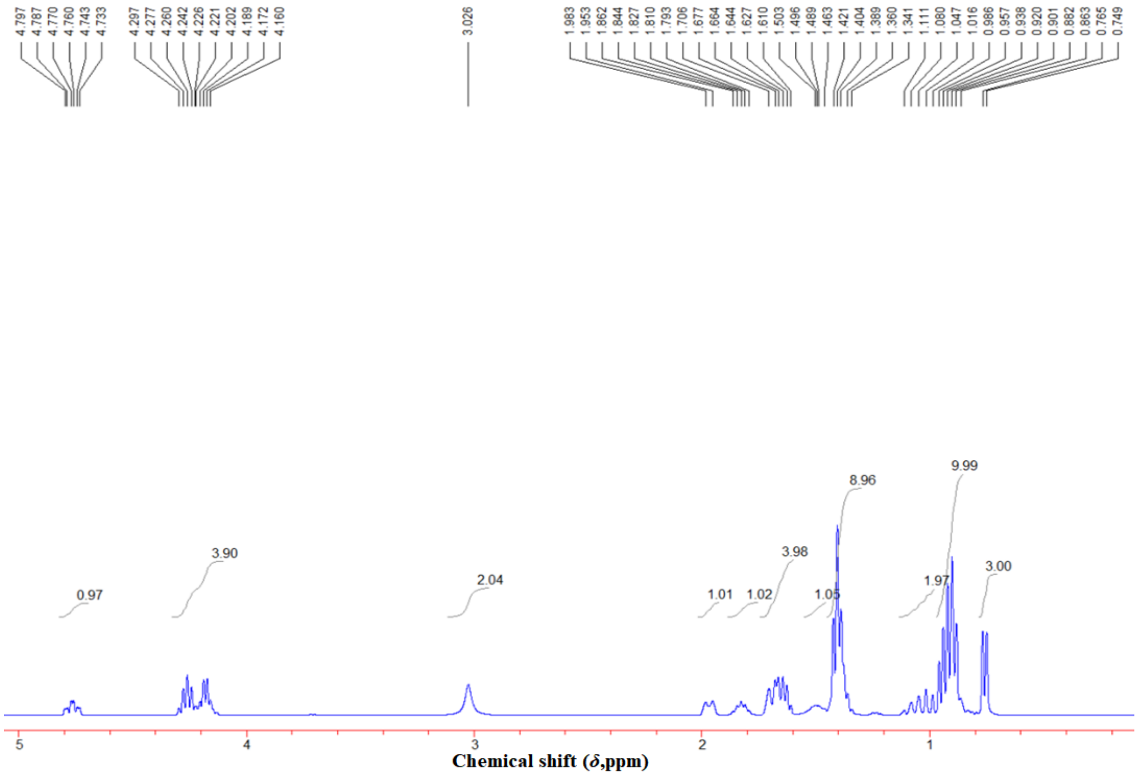

Figure S1. The ^{1}H NMR spectra of ML-BL-1 (400 MHz, $CDCl_3$).

Chemical shift (δ, ppm): 0.749-0.765 (d, 3 H), 0.863-0.957 (m, 10 H), 0.986-1.111 (m, 2 H), 1.341-1.421 (m, 9 H), 1.463-1.503 (m, 1 H), 1.610-1.706 (m, 4 H), 1.793-1.862 (m, 1 H), 1.953-1.983 (d, 1 H), 3.026 (s, 2 H) ,4.160-4.297 (m, 4 H), 4.733-4.797 (m, 1H).

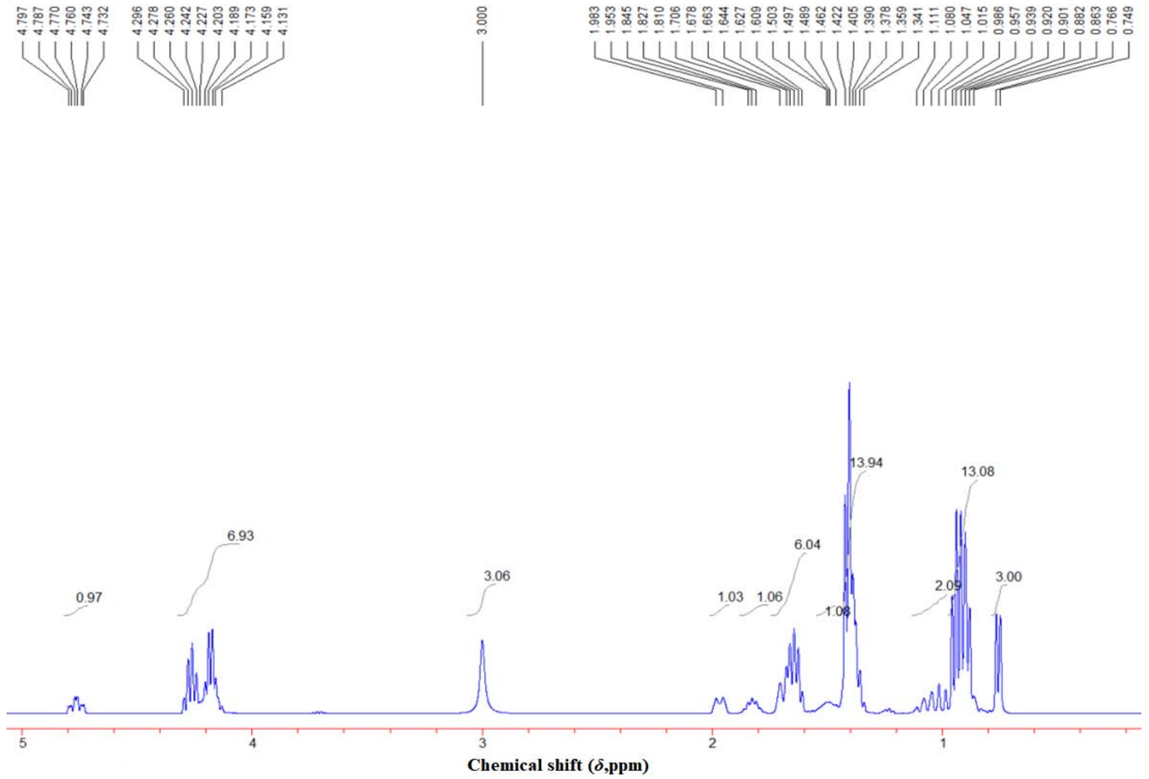

Figure S2. The ^{1}H NMR spectra of ML-BL-2 (400 MHz, $CDCl_3$).

Chemical shift (δ, ppm): 0.749-0.766 (d, 3 H), 0.863-0.957 (m, 13 H), 0.986-1.111 (m, 2 H), 1.341-1.422 (m, 14 H), 1.462-1.503 (m ,1 H), 1.609-1.706 (m, 6 H), 1.810-1.845 (m, 1 H), 1.953-1.983 (d, 1 H), 3.000 (s, 3 H), 4.131-4.296 (m, 7 H), 4.732-4.797 (m, 1 H).

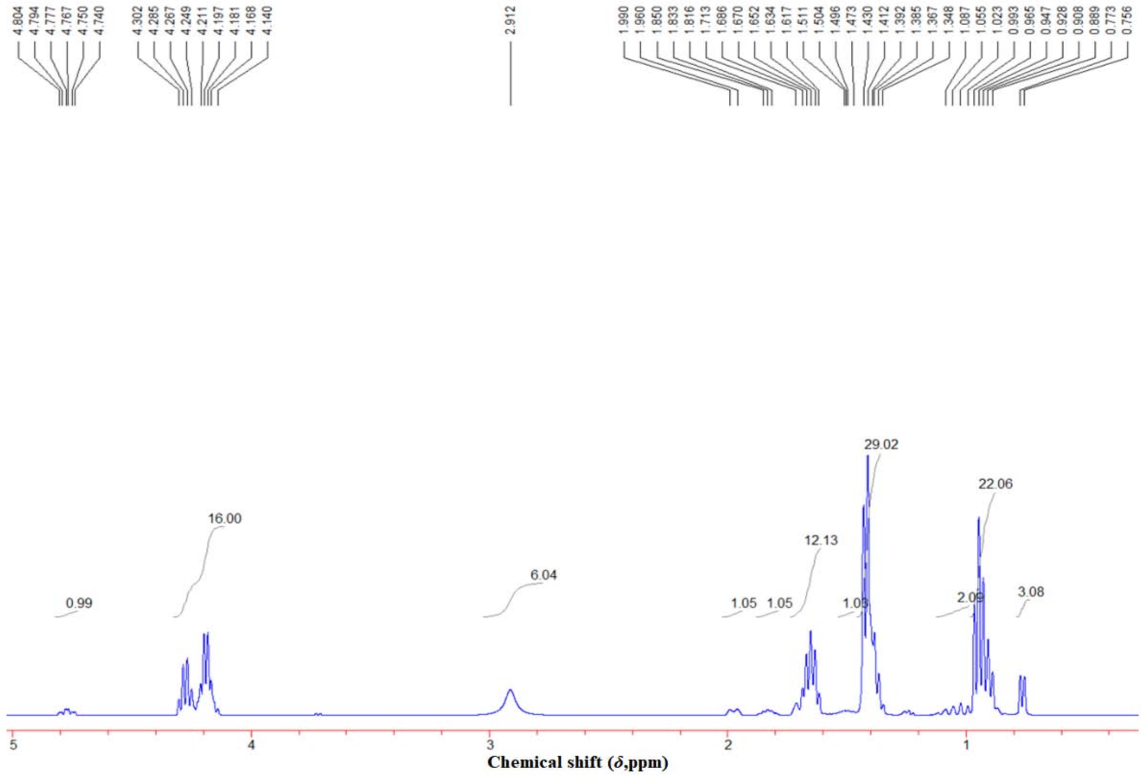

Figure S3. The ^{1}H NMR spectra of ML-BL-3 (400 MHz, $CDCl_3$).

Chemical shift (δ, ppm): 0.756-0.773 (d, 3 H), 0.889-0.965 (m, 22 H), 0.993-1.087 (m, 2 H), 1.348-1.430 (m, 29 H), 1.473-1.511 (m,1 H), 1.617-1.713 (m, 12 H), 1.816-1.850 (m, 1 H), 1.960-1.990 (d, 1 H), 2.912 (s, 6 H), 4.140-4.302 (m, 16 H), 4.740-4.804 (m, 1 H).

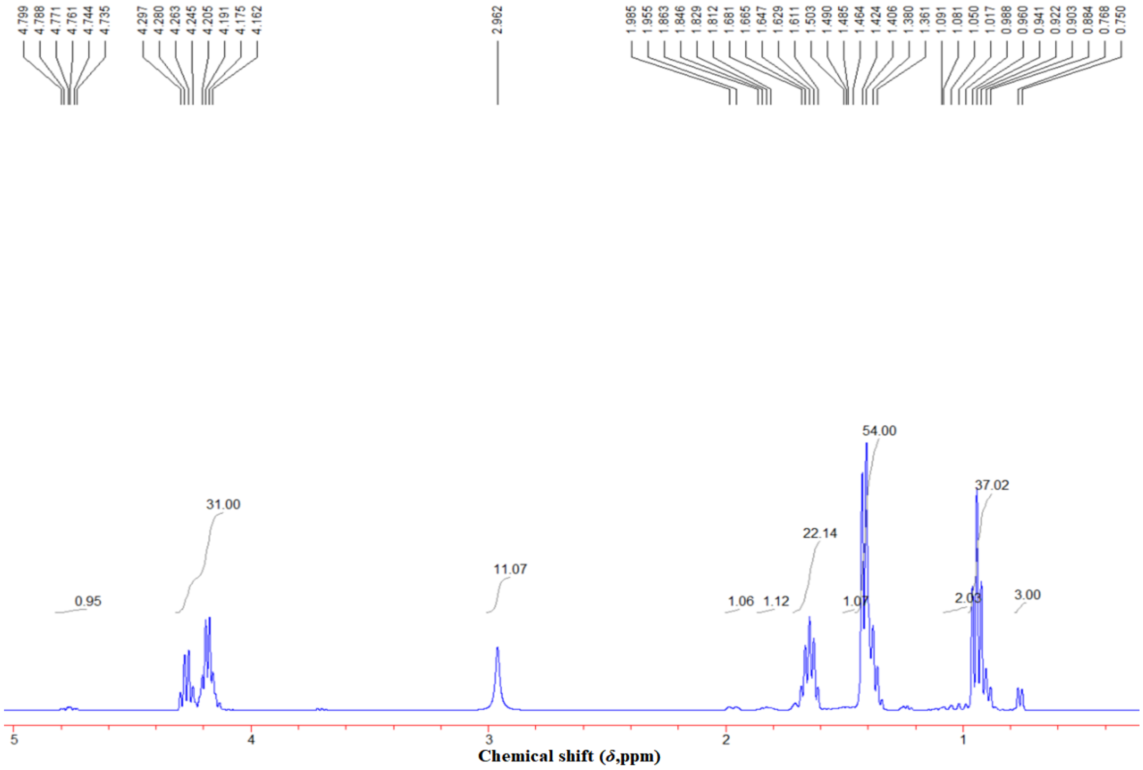

Figure S4. The ^{1}H NMR spectra of ML-BL-4 (400 MHz, $CDCl_3$).

Chemical shift (δ, ppm):0.750-0.768 (d, 3 H), 0.884-0.960 (m, 37 H), 0.988-1.091 (m, 2 H), 1.361-1.424 (m, 54 H), 1.464-1.503 (m,1 H), 1.611-1.681 (m, 22 H), 1.812-1.863 (m, 1 H), 1.955-1.985 (d, 1 H), 2.962 (s, 11 H), 4.162-4.297 (m, 31 H), 4.735-4.799 (m, 1 H).

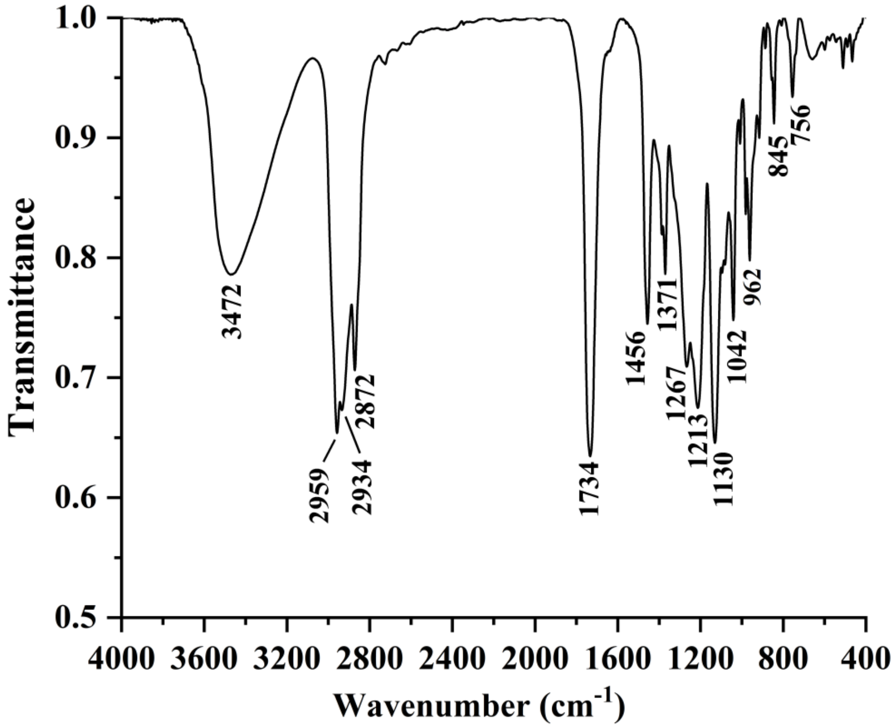

Figure S5. The FT-IR spectra of ML-BL-1.

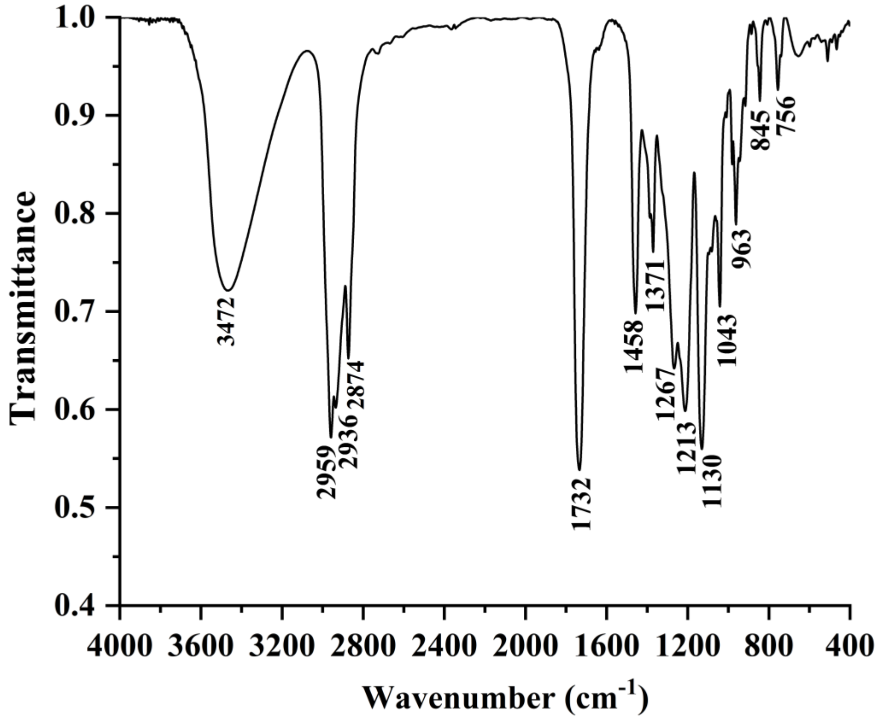

Figure S6. The FT-IR spectra of ML-BL-2.

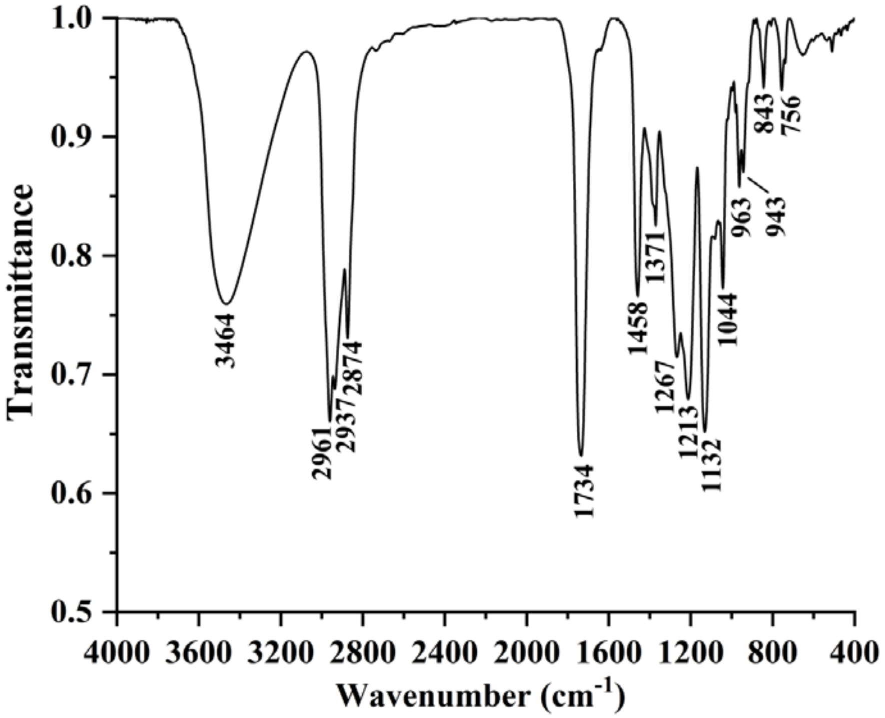

Figure S7. The FT-IR spectra of ML-BL-3.

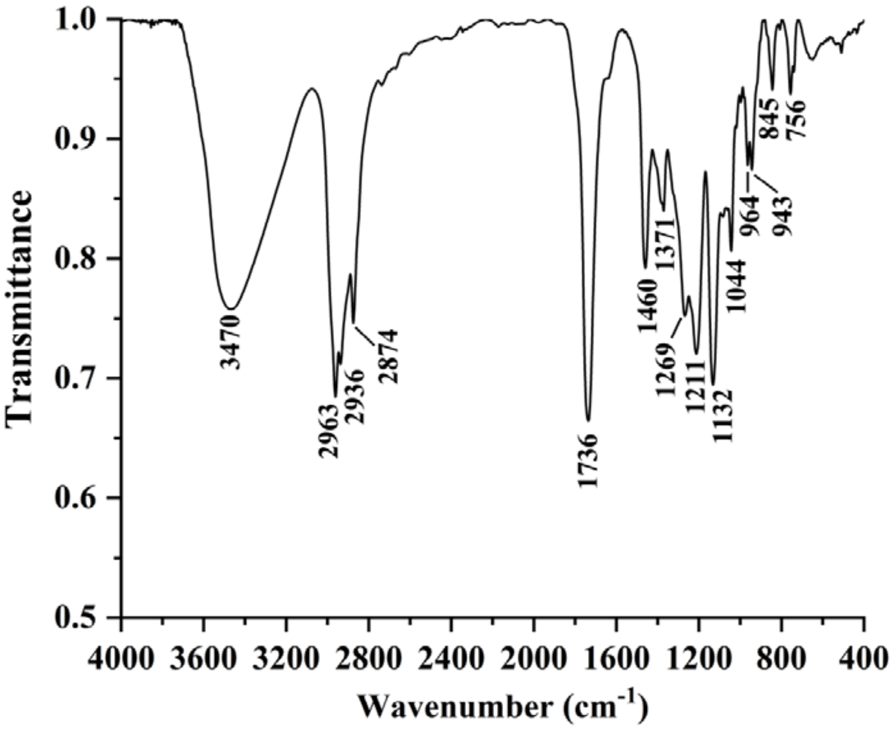

Figure S8. The FT-IR spectra of ML-BL-4.

Section S2. Identification of Hydrogen Bond Donors (HBDs) and Hydrogen Bond Acceptors (HBAs) in BNDESs

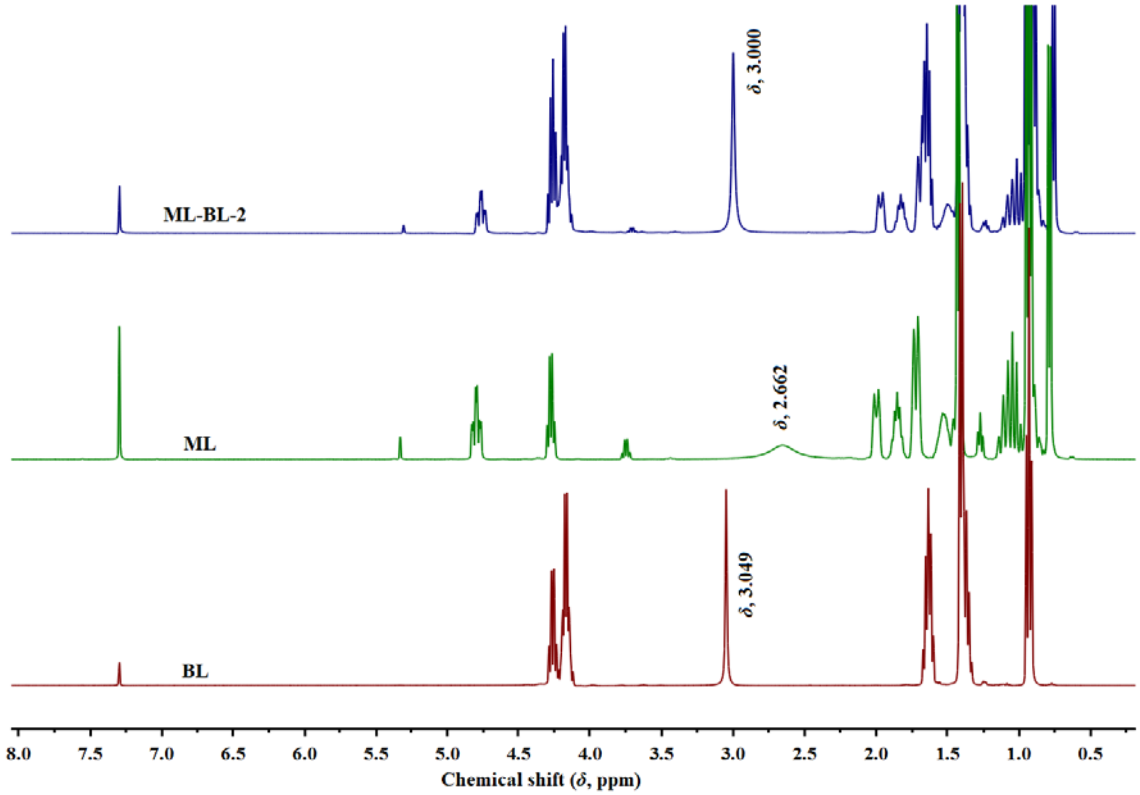

Figure S9. The ^{1}H NMR spectra of BL, ML and ML-BL-2 (400 MHz, $CDCl_3$). Chemical shifts marked in the figure are the hydroxyl proton signals of the corresponding compounds.

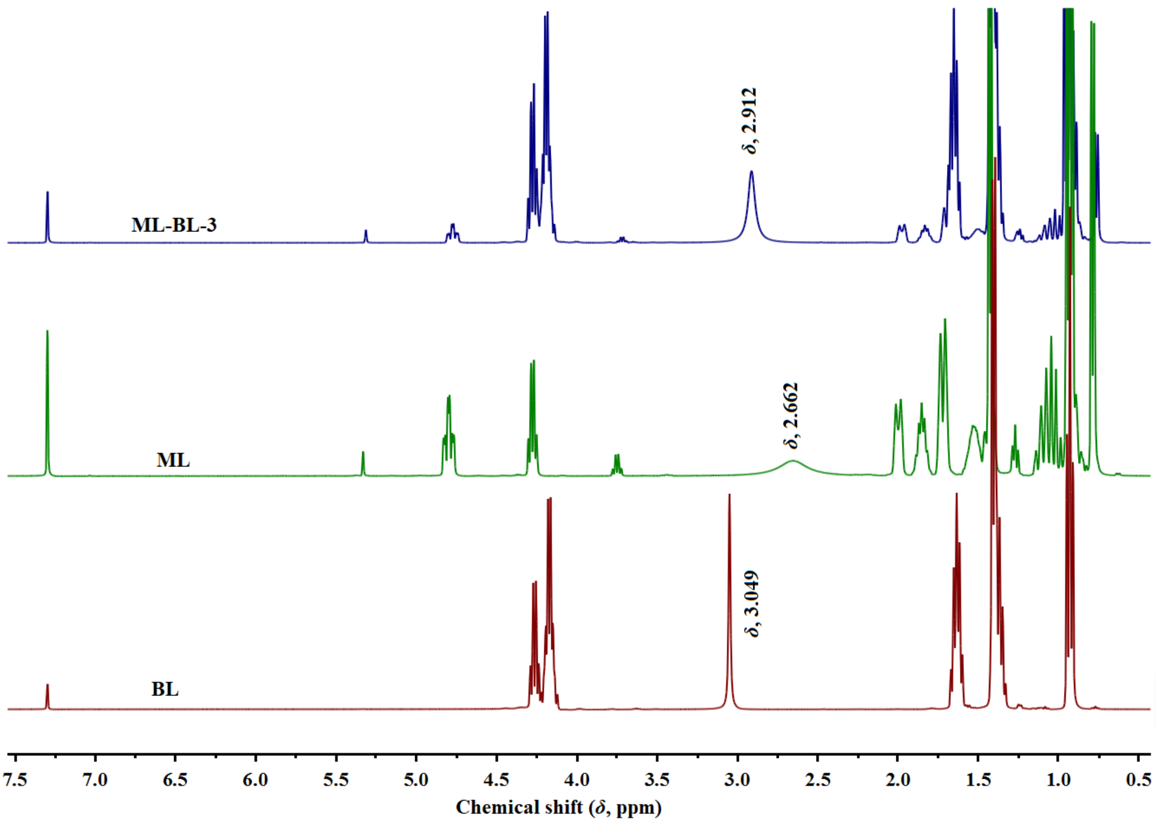

Figure S10. The ^{1}H NMR spectra of BL, ML and ML-BL-3 (400 MHz, $CDCl_3$).Chemical shifts marked in the figure are the hydroxyl proton signals of the corresponding compounds.

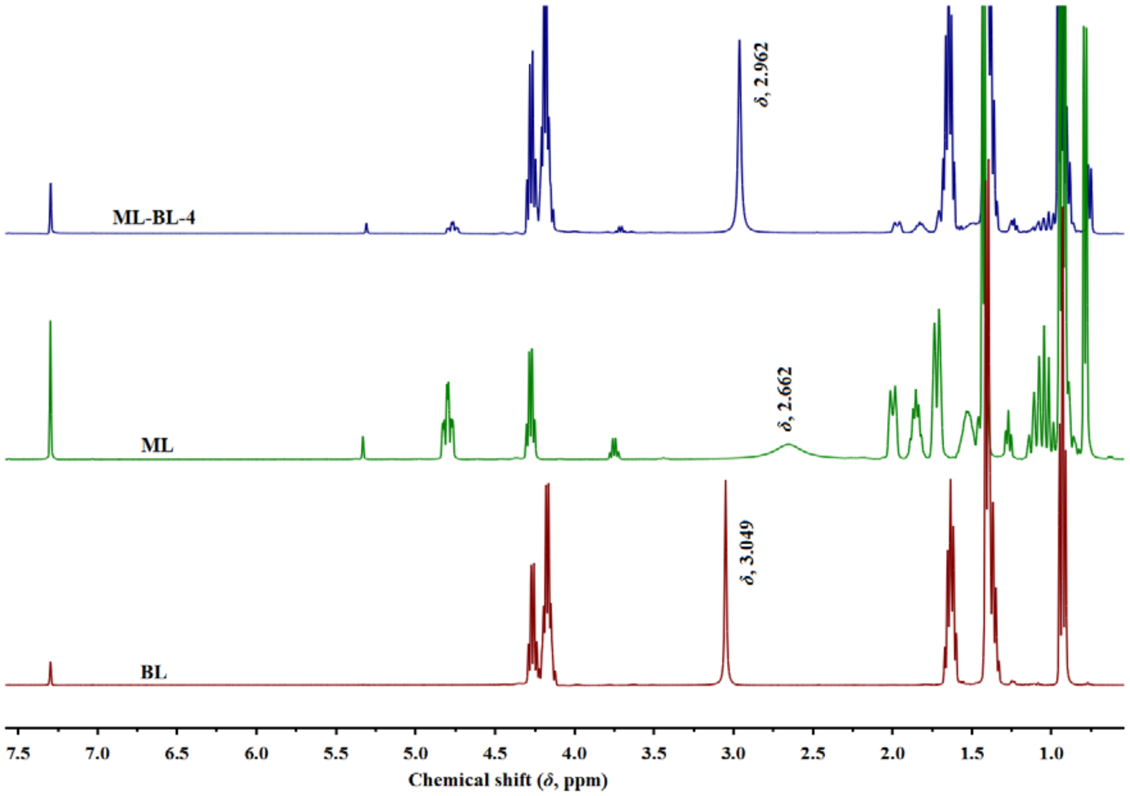

Figure S11. The ^{1}H NMR spectra of BL, ML and ML-BL-4 (400 MHz, $CDCl_3$).Chemical shifts marked in the figure are the hydroxyl proton signals of the corresponding compounds.

Section S3. Optimization of the SPE and LLE Conditions

As illustrated in Figure S12, the adsorption of CA and RA on CAD-40 and HP-20 macroporous resins reaches equilibrium within 20 min and 30 min, respectively. Therefore, 20 min and 30 min are selected as the optimal adsorption time for CAD-40 and HP-20 macroporous resins, respectively.

It can be seen from Figure S13a and Figure S14a, CAD-40 exhibits better adsorption selectivity for CA compared with HP-20 and a solid-liquid ratio of 0.1 g mL^{-1} for the selective adsorption of CA from the PEG-400 extract by CAD-40 is a good choice in combination of adsorption efficiency and selectivity. Meanwhile, Figure S13b and Figure S14b suggest that the adsorption capacity of CAD-40 and HP-20 for RA is still low even diluting the PEG-400 extract 4-fold. Additionally, Figures S15–S17 demonstrate that the optimal extraction time for the extraction of RA and CA from the PEG-400 extract by EA, ML-BL-3(a representative of the four ML-BL-based BNDESs) and [C_4mim]PF_6 is 1 min, 5 s and 5 min, respectively.

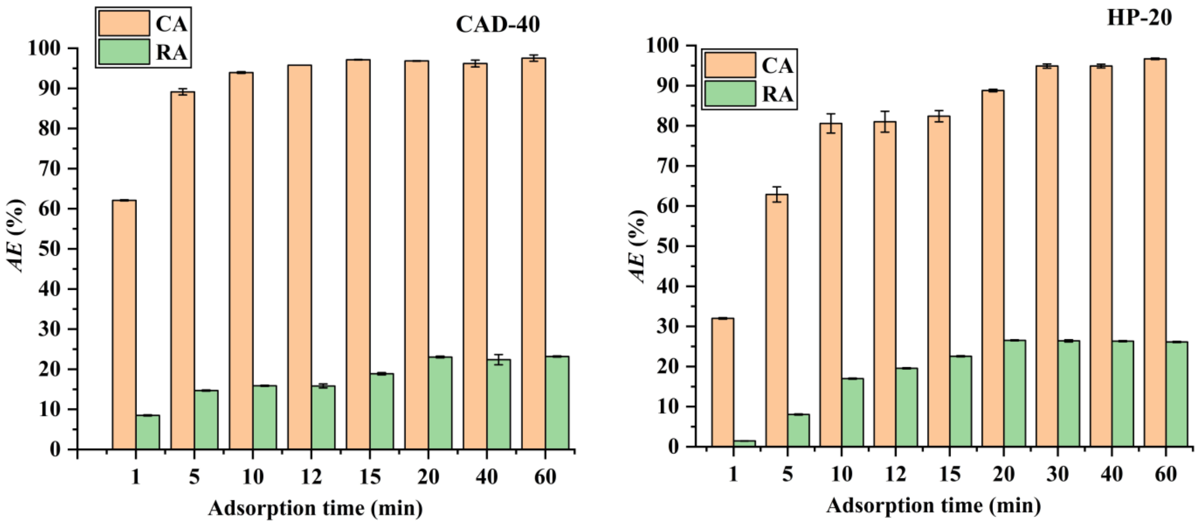

Figure S12. Effect of time on the adsorption of CA and RA on CAD-40 and HP-20 macroporous resins from the PEG-400 extract. Solid-liquid ratio, 0.1 g mL^{-1}; adsorption temperature, 25 ^{o}C.

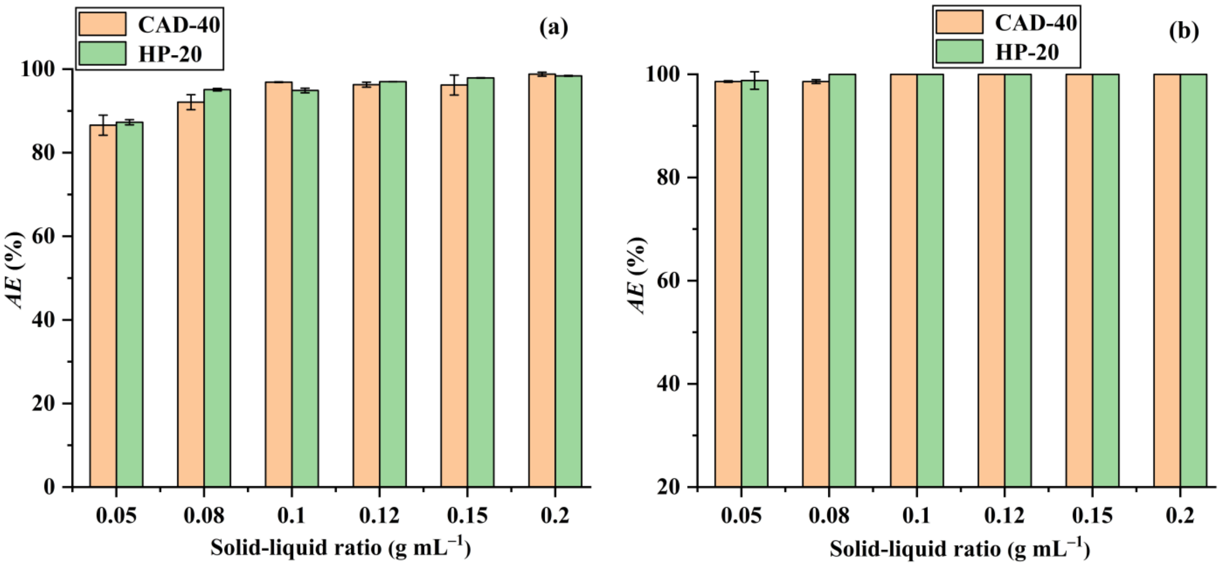

Figure S13. Effect of solid-liquid ratio on the adsorption of CA on CAD-40 and HP-20 macroporous resins from the PEG-400 extract (a) and the PEG-400 extract diluted 4-fold (b). Adsorption temperature, 25 ^{o}C.

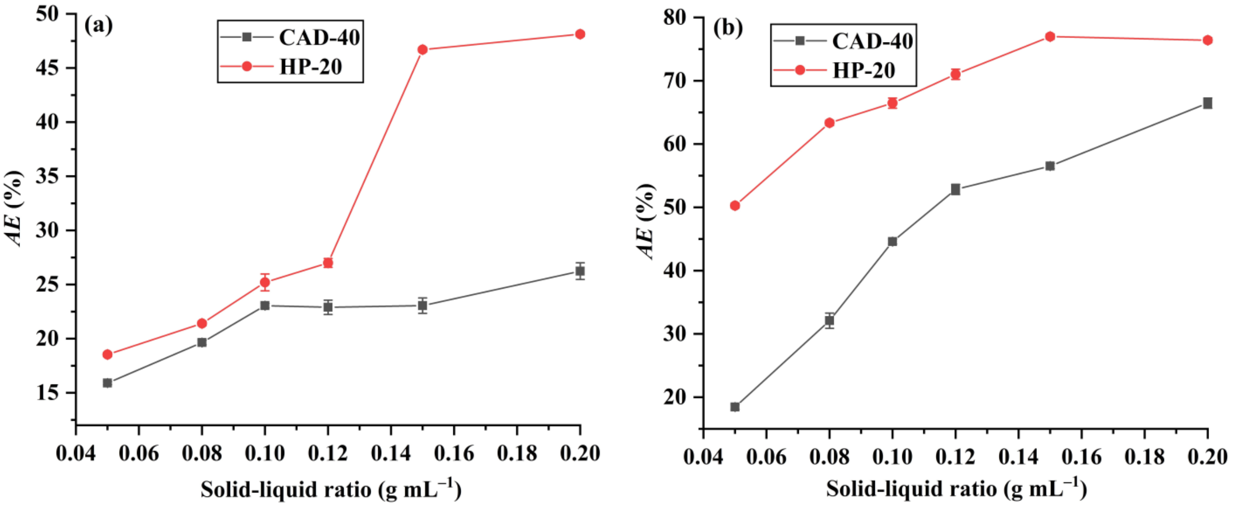

Figure S14. Effect of solid-liquid ratio on the adsorption of RA on CAD-40 and HP-20 macroporous resins from the PEG-400 extract (a) and the PEG-400 extract diluted 4-fold (b). Adsorption temperature, 25 ^{o}C.

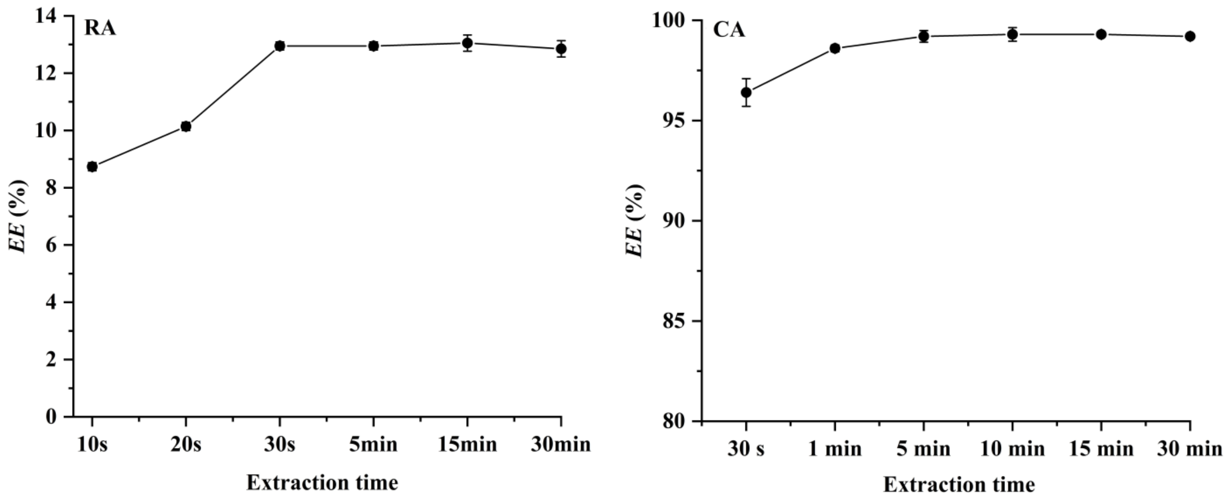

Figure S15. Effect of time on the extraction of RA and CA by EA from the PEG-400 extract. Extraction temperature, 25 °C; V_{EA} : $V_{\mathrm{PEG\text{-}400\ extract}}$ = 1 : 1.

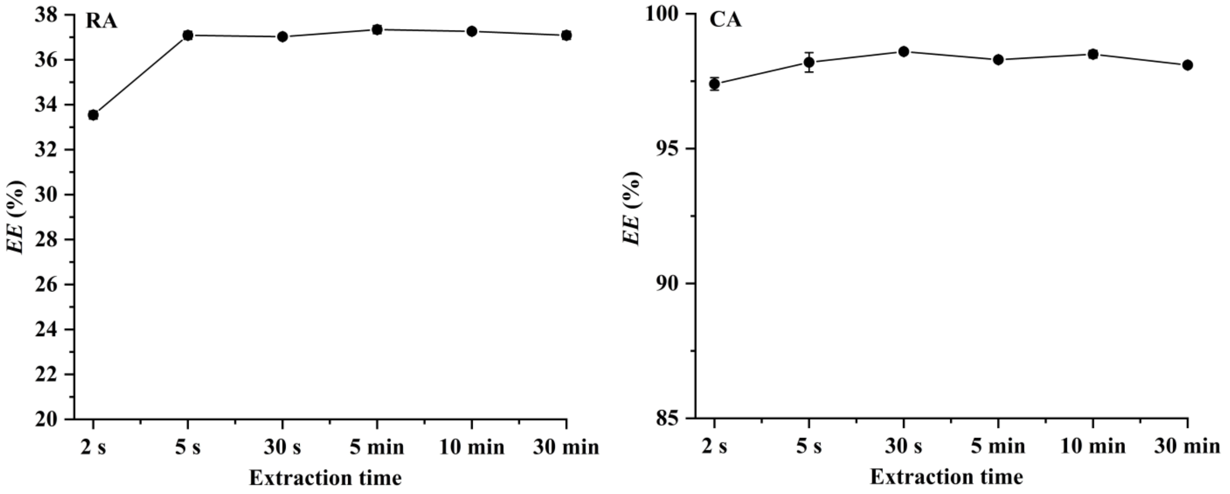

Figure S16. Effect of time on the extraction of RA and CA by ML-BL-3 from the PEG-400 extract. Extraction temperature, 25 °C; $V_{\mathrm{DES\text{-}3}}$: $V_{\mathrm{PEG\text{-}400\ extract}}$ = 1 : 1.

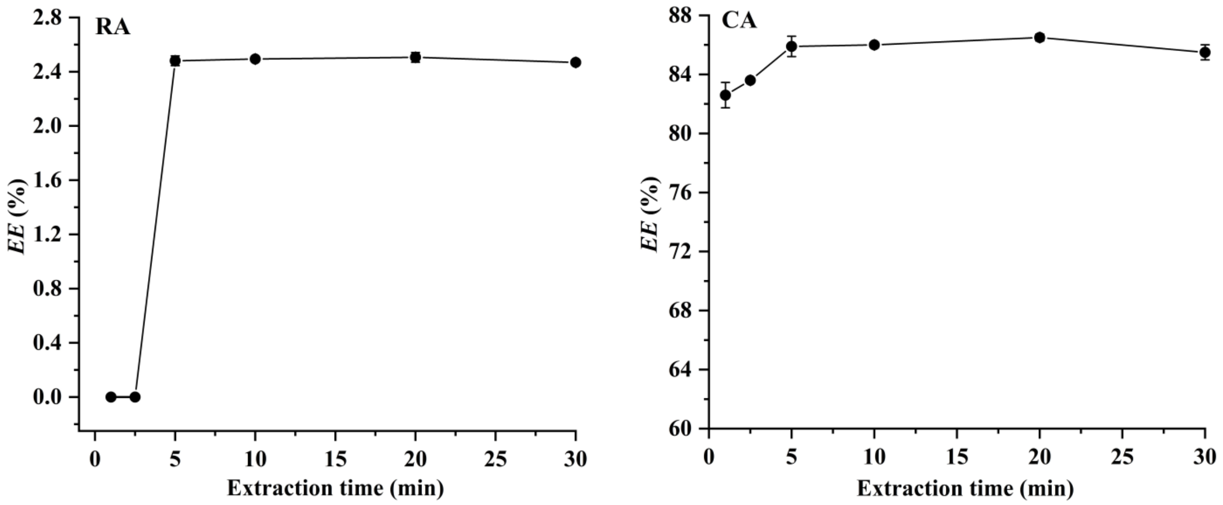

Figure S17. Effect of time on the extraction of RA and CA by the IL [C_4mim]PF_6 from the PEG-400 extract. Extraction temperature, 25 ^oC; V_{IL} : $V_{PEG\text{-}400\ extract}$ = 1 : 1.
